# Disentangling the Molecular Mechanisms of the Antidepressant Activity of Omega-3 Polyunsaturated Fatty Acid: A Comprehensive Review of the Literature

**DOI:** 10.3390/ijms22094393

**Published:** 2021-04-22

**Authors:** Hans O. Kalkman, Martin Hersberger, Suzanne Walitza, Gregor E. Berger

**Affiliations:** 1 Child and Adolescent Psychiatry Research Centre, Department of Child and Adolescent Psychiatry and Psychotherapy, Psychiatric University Hospital, University of Zurich, CH-8032 Zurich, Switzerland; suzanne.walitza@pukzh.ch; 2 Division of Clinical Chemistry and Biochemistry, University Children’s Hospital Zurich, CH-8032 Zurich, Switzerland; Martin.Hersberger@kispi.uzh.ch; 3 Children’s Research Center, University Children’s Hospital Zurich, CH-8032 Zurich, Switzerland; 4 Center for Integrative Human Physiology, University of Zurich, CH-8057 Zurich, Switzerland

**Keywords:** ω-3 polyunsaturated fatty acid, EPA-paradox, resolvin, cytochrome P450 isoenzyme, epoxide, endocannabinoid, CB2 receptor

## Abstract

Major depressive disorders (MDDs) are often associated with a deficiency in long-chain omega-3 polyunsaturated fatty acids (ω-3 PUFAs), as well as signs of low-grade inflammation. Epidemiological and dietary studies suggest that a high intake of fish, the major source of ω-3 PUFAs, is associated with lower rates of MDDs. Meta-analyses of randomized placebo-controlled ω-3 PUFAs intervention-trials suggest that primarily eicosapentaenoic acid (EPA), but not docosahexaenoic acid (DHA), is responsible for the proposed antidepressant effect. In this review, we dissect the current biological knowledge on EPA and DHA and their bioactive lipid metabolites to search for a pharmacological explanation of this, to date, unexplained clinical observation. Through enzymatic conversion by cyclooxygenase (COX), lipoxygenase (ALOX), and cytochrome P-450 monooxygenase (CYP), EPA and DHA are metabolized to major anti-inflammatory and pro-resolving lipid mediators. In addition, both ω-3 PUFAs are precursors for endocannabinoids, with known effects on immunomodulation, neuroinflammation, food intake and mood. Finally, both ω-3 PUFAs are crucial for the structure and organization of membranes and lipid rafts. While most biological effects are shared by these two ω-3 PUFAs, some distinct features could be identified: (1) The preferential CYP monooxygenase pathway for EPA and EPA derived eicosanoids; (2) The high CB2 receptor affinities of EPA-derived EPEA and its epoxy-metabolite 17,18-EEQ-EA, while the DHA-derived endocannabinoids lack such receptor affinities; (3) The competition of EPA but not DHA with arachidonic acid (AA) for particular glycerophospholipids. EPA and AA are preferentially incorporated into phosphatidylinositols, while DHA is mainly incorporated into phosphatidyl-ethanolamine, -serine and -choline. We propose that these distinct features may explain the superior antidepressant activity of EPA rich ω-3 PUFAs and that these are potential novel targets for future antidepressant drugs.

## 1. Introduction

Epidemiological studies report an inverse association between intake of oily fish and the prevalence [1,2,3,4,5,6] and incidence of major depressive disorders (MDDs)* [7,8,9]. Greater seafood consumption also predicts lower prevalence rates for postpartum depression [10] and lower lifetime prevalence rates for bipolar I disorder, bipolar II disorder, and bipolar spectrum disorders [11]. A recent meta-analysis of 26 studies involving 150,278 participants found that high versus low fish intake protects against depression with a pooled relative risk of 0.83 [12].

In line with the lower seafood consumption, lower ω-3 PUFA levels have been measured in erythrocyte cell membranes or plasma of adult MDD patients [13], as well as in mothers with postpartum depression [14]. A meta-analytic review including 684 patients with MDD and 2670 healthy controls [15] found significantly lower levels of ω-3 PUFAs in the erythrocyte-membranes of MDD patients (Effect Size [ES] = −0.51, *p* < 0.0001), in particular of EPA (ES = −0.18, *p* = 0.004) and DHA (ES = −0.35, *p* = 0.0002). Lower levels of ω-3 PUFAs lead to increased ω-6/ω-3 ratios frequently reported in adult MDD [16,17,18,19], as well as in drug-naive pediatric MDD [20]. In addition, independent groups reported inverse associations between membrane ω-3 PUFAs and the number of suicide attempts [2,21,22].

The epidemiological inverse association between fish intake and depression, as well as the observation of low ω-3 PUFAs in erythrocyte membranes of patients with MDD, triggered a range of ω-3 PUFAs intervention trials. Most of these studies were of a small scale; however, in most of these randomized placebo-controlled trials (RCTs) a beneficial effect of ω-3 PUFAs on depressive symptoms was corroborated across the lifespan. In recent years, these small-scale RCTs have been evaluated in several meta-analyses, [23,24,25,26,27,28,29,30], which, however, differed in their inclusion criteria (e.g., combining clinical with non-clinical populations, see Table 1). Beneficial effects of ω-3 PUFAs on MDD were observed in all but one meta-analyses [29] and yielded standardized mean differences (SMD) of 0.22–0.56 for primary and secondary depression [23,24,25]. The one meta-analysis that did not observe a beneficial effect of ω-3 PUFAs on MDD [29] included RCTs, in which the criteria for clinical depression were not met. It is likely that the unrestricted Bloch and Hannestad meta-analysis was confounded by a single large study [31], which investigated the antidepressant effects of ω-3 PUFAs on mild depressive symptoms in a large non-clinical population. Indeed, when the same authors restricted their meta-analysis to RCTs only including patients meeting criteria for MDD, they observed a moderate beneficial effect for ω-3 PUFAs with a SMD of 0.42 [29].

Furthermore, ω-3 PUFA composition and doses varied substantially between RCTs included in previous meta-analyses [23,24,25,26], increasing the risk for a negative overall effect in case that only one particular ω-3 PUFA has an antidepressant effect [23,24,25,27,28,29,30]. While 21 studies, using supplements containing greater than 50% EPA or pure ethyl-EPA, observed a significant reduction in depressive symptoms [23], studies using purified DHA or PUFA compositions containing more than 50% DHA showed no beneficial effect [32,97,98]. Two more recent meta-analyses [24,26] confirmed this early finding. Hence, the results from the available RCTs suggest that EPA-rich, rather than DHA-rich formulations have an antidepressant effect, particularly in primary MDD [23,24,25,26]. In this review, we, therefore, investigate the current knowledge about the biology of EPA, DHA, and of their metabolites in order to find a pharmacological explanation for the proposed superior antidepressant effects of EPA. (* A list of abbreviations is provided at the end of the article.)

## 2. Omega-3 PUFAs and Immunomodulation

### 2.1. Anti-Inflammatory Effects of EPA and DHA

Several lines of evidence support an altered immune-status in depression, with pro-inflammatory cytokines in both plasma and cerebrospinal fluid influencing the progression and severity of depressive disorders [99,100,101,102,103,104,105,106,107]. While ω-3 PUFAs have been shown to reduce the secretion of pro-inflammatory cytokines in several cellular and animal models [108], there is also increasing support for a similar effect in humans. Two RCTs in conditions with chronic inflammation and depression demonstrated a protective effect of ω-3 PUFAs against the development of depressive symptoms, which correlated with a decrease in pro-inflammatory mediators [109,110]. These associations of reduced inflammation with better clinical outcome were further substantiated by an RCT showing that MDD patients with higher levels of inflammatory markers benefitted more from ω-3 PUFAs, than patients with no signs of low-grade inflammation [33]. This finding suggests that at least some of the pharmacological effects of the ω-3 PUFAs in MDD may be mediated through an anti-inflammatory mechanism.

EPA and DHA share a number of pharmacological properties that contribute to the suppression of inflammation [108,111,112]. They both act as competitive antagonists at the Toll-like receptor-4 (TLR4), which mediates the pro-inflammatory activity of saturated fatty acids [113] and lipopolysaccharides [114], and they activate the transcription factor PPARγ [115]. The latter suppresses the activity of the pro-inflammatory NFκB signaling-cascade [116]. DHA seems to be more potent in inhibiting NFκB signaling [108,115,117], as well as in enhancing intracellular levels of glutathione, reducing nitric-oxide production [118], and reducing the expression of COX2 [119], while EPA seems to be more powerful in reducing the expression of IL-1β and the chemokine MCP-1 [119]. The mechanism leading to IL-1β secretion is a well-controlled mechanism, where cells recognize the presence of danger signals [120,121] and respond by mounting an immune response. This immune response is initiated by the formation of a multi-protein complex called inflammasome, and results in the production and secretion of active IL-1β (reviewed by [121]).

In case macrophages or microglia are confronted with a danger signal (e.g., stress reaction, viral infections), they assemble an inflammasome called NLRP3, whereas neurons generate an inflammasome with a slightly different composition, called NLRP1 [122,123]. Alcocer-Gómez et al. [124] observed increased gene expression of NLRP3 and caspase-1 in blood cells, and increased serum levels of IL-1β and IL-18 in non-treated patients with adult MDD, whilst IL-1β and IL-18 correlated with the Beck Depression Inventory scores [124]. In addition, in a recent study including 299 depressed Vietnam War veterans [125], carriers of the NLRP12 polymorphisms (rs34436714) were associated with a higher DASS21 Score for depression (*p* = 0.037).

It has been shown that saturated fatty acids induce inflammasomes [121] and support inflammation [126], whereas DHA and EPA suppress the generation of inflammasomes, probably through G protein-coupled receptor signaling (GPR120 and GPR40), ultimately inhibiting the IL-1β secretion [126].

Reactive oxygen species (ROS) represent another trigger for the induction of inflammasomes [127] and there is indication from cell culture experiments that ω-3 PUFAs reduce ROS formation [128,129]. Proton magnetic resonance spectroscopy enables measurement of the intracellular antioxidant glutathione in the living human brain [130] that protects cells from the oxidative damage associated with increased ROS formation. Adults at risk of depression showed an attenuated glutathione/creatinine ratio that inversely correlated with an increase in depressive symptom severity [131]. In another study in first-episode psychosis patients, twelve weeks treatment with ethyl-EPA supplementation led to a marked increase in glutathione of more than 20% that closely correlated with an improvement in negative symptoms [132]. The modulation of the intracellular redox balance by ω-3 PUFAs may, therefore, be one potential mechanism of how ω-3 PUFAs modulate inflammation and promote neuroprotection [133], possibly by inhibiting the production of the generation of NLRP3 inflammasomes via Redox Balance Modulation [134].

In summary, there is compelling evidence that ω-3 PUFAs, in particular, EPA and DHA, suppress pro-inflammatory and promote anti-inflammatory pathways. Both ω-3 PUFAs suppress NFκB signaling, inhibit inflammasome formation, down-regulate cyclooxygenase-2 transcription and counteract redox misbalances. Although both ω-3 PUFAs have preferences in their affinity with particular inflammatory signaling cascades, EPA seems to be more potent in reducing IL-1β and chemokine MCP-1 production and, therefore, inhibiting inflammasome production. However, at this stage, it would be premature to balance these differential effects against each other and to classify either DHA or EPA as the stronger anti-inflammatory effector.

### 2.2. Pro- and Anti-Inflammatory Oxidation Products from EPA and DHA

#### 2.2.1. Prostaglandins and Leukotrienes

**Figure 1:**
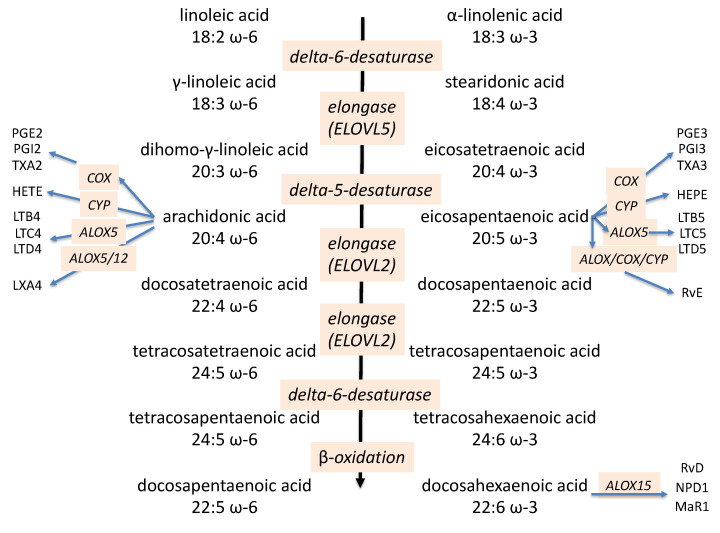
Omega-3/omega-6 PUFA pathways and their bioactive lipid metabolites.

The released PUFAs form a pool of precursors that are metabolized to distinct bioactive lipid mediators by three major pathways: the cyclooxygenase (COX) pathway, the lipoxygenase pathway (ALOX), and the cytochrome P-450 monooxygenase (CYP) pathway (Figure 1; for an extensive review, see [135]). The products from PUFAs of the ω-6 and ω-3 families are also called oxylipins. The exact profile and balance of bioactive products derived from this PUFA pool depends on cell and tissue type and is determined by environmental and physiological contexts.

The enzymatic oxidation of AA, EPA and DHA by these three major pathways gives rise to a wide spectrum of bioactive lipid mediators, which are called “eicosanoids” when metabolized from AA and EPA and docosanoids when derived from DHA. At the beginning of inflammation, the COX and the ALOX5 enzymes, respectively, generate pro-inflammatory 2-series prostaglandins (PG…2) and the 4-series leukotrienes (LT…4) from AA. This enzymatic conversion is competitively inhibited by both EPA and DHA, which limits the inflammatory effect of the AA derived eicosanoids. Notably, EPA is itself metabolized by the COX and ALOX5 enzymes to generate the 3-series prostaglandins and 5-series leukotrienes. These EPA-derived 3-series prostaglandins and 5-series leukotrienes were shown to be partial agonists of the same receptors triggered by the AA-derived 2-series prostaglandins and 4-series leukotrienes, and it is thought that they competitively suppress the pro-inflammatory triggering of the AA-derived eicosanoids. For example, the AA-derived LTB4 is a potent chemo-attractant for neutrophils, eosinophils and macrophages, whereas the EPA-derived LTB5 analogue acts as partial agonist only [136,137,138]. While both ω-3 PUFAs inhibit the conversion of AA to the mainly pro-inflammatory eicosanoids, only EPA gives rise to metabolites, which are weak agonists at certain receptors that mediate pro-inflammatory responses of AA derived 2-series prostaglandins and 4-series leukotrienes [115].

#### 2.2.2. Resolvins and Other Poly-Hydroxyl Products

While the prostaglandins and leukotrienes depend on COX and ALOX5 enzymatic activities, other classes of lipid mediators, called specialized pro-resolving lipid mediators (SPMs), like the resolvins, maresins, and neuroprotectins, depend on enzymatic oxygenation by CYP450 and ALOX12/15 [139]. For example, the E-series of the resolvins derive from EPA, which is oxidized to dihydroxy-EPA derivatives (RvE2 and RvE3) [140,141] and a tri-hydroxy-EPA derivative (RvE1) [142]. Similarly, the metabolism of DHA generates several di-hydroxy and tri-hydroxy derivatives, referred to as maresins, neuroprotectin-D and D-series of resolvins (for reviews, see [143,144]).

These SPMs diminish inflammation by reducing the influx of neutrophils to inflamed tissue and promote the resolution of inflammation by increasing the phagocytic activity of monocytes and macrophages [143,145]. Application of these SPMs, such as RvD1 or RvE1, in mouse models of chronic inflammatory diseases consistently induced anti-inflammatory and pro-resolution effects [146]. Intriguingly, RvE1 and RvD1 suppressed inflammation and depression-like behavior in animal depression models [147,148,149,150] and in several animal models of chronic inflammatory diseases that are associated with major depression, like periodontitis, type II diabetes and atherosclerosis [151,152,153]. Nevertheless, judged by data from preclinical experiments, there is little evidence to date for a fundamental distinction between EPA- and DHA-derived mediators [154], although minor differences have been described [155,156,157]. Future research will have to dissect differences in the pharmacology of EPA- and DHA-derived SPMs to address differences in their antidepressant properties.

Microglial cells are the brain’s innate immune cells and contribute to the shaping of neuronal networks during brain development. Rey et al. [158] found that microglial cells in the offspring of pregnant mice fed with deficient, balanced or supplemented ω-3 PUFA diets, displayed distinctive lipid profiles, with higher levels of EPA than DHA. The same group [159] showed that dietary ω-3 PUFA supplementation induced ω-3 PUFA enrichment in the hippocampus which led to an increase in ω-3 PUFA-derived oxylipins and a decrease in ω-6 PUFA-derived oxylipins upon LPS stimulation. In addition, the LPS-induced pro-inflammatory cytokine increase was reduced by dietary ω-3 PUFA supplementation. These results indicate that brain ω-3 PUFAs promote the synthesis of anti-inflammatory oxylipins.

#### 2.2.3. CYP Metabolism of Double Bonds or the Terminal Carbon Atom

The third oxidation pathway leading to bioactive eicosanoids and docosanoids is mediated by certain cytochrome P450 isoenzymes (CYPs), including CYP1A1, CYP2E1, CYP4A1 and CYP4A12a. These CYPs hydroxylate AA at its terminal ω1-carbon forming 20-HETE, which has pro-inflammatory, vasoconstrictive and hypertensive properties [160,161]. The same isoenzymes, however, generate an epoxide at the ω-3 double bond of EPA and DHA [162,163,164], producing 17,18-EEQ and 19,20-EDP, respectively, which display vasodilatory and anti-inflammatory activities in the cardiovascular system, the bronchi, kidney and the nervous system [165,166]. The receptor for 20-HETE has recently been identified as GPR75, but it has not been investigated whether the two ω-3 PUFA-derived lipid mediators, 17,18-EEQ and 19,20-EDP, compete for binding to GPR75 [167]. This may become interesting because ω-3 PUFA supplementation has a pronounced effect on cellular AA levels [164,168,169], shifts the CYP mediated lipid mediators from predominantly AA-metabolites to EPA- and DHA-derived metabolites, and increases the plasma and tissue levels of 17,18-EEQ and 19,20-EDP [168]. There is indication that this ω-3 PUFA supplementation-mediated shift in the CYP-metabolome mainly produces EPA-metabolites, and that this increase is more pronounced than for the COX- and ALOX-metabolomes [168].

#### 2.2.4. Epoxidation of Endocannabinoids

Both series of PUFAs are also substrates for the endogenous production of endocannabinoids. The endocannabinoid system is known to be involved in numerous functions such as appetite control, food intake, energy balance, neuroprotection, neurodegenerative diseases, mood disorders, modulation of pain, and inflammatory responses [170]. The best-known endogenous ligands for the two main cannabinoid-receptors CB1 and CB2 are the AA-derived anandamide (arachidonic acid-ethanolamide; AEA) and 2-arachidonyl-glycerol [171,172]. However, also EPA and DHA are metabolized to ethanolamides, resulting in compounds known as EPEA and DHEA, respectively [173,174]. All endocannabinoids are further metabolized by COX2, ALOX and CYPs, resulting in diverse active bioactive lipid metabolites with different selectivities for the different cannabinoid-receptors [174,175,176]. For example, CYP mediated epoxidation of the potent and selective CB1 agonist anandamide [174,177], and generates the selective CB2 receptor agonist 5,6-EET-EA [175]. In contrast to the AA-derived anandamide, the EPA metabolite EPEA displays high affinity for both cannabinoid receptors, while epoxidation of the susceptible ω-3 double bond yields 17,18-EEQ-EA with weak CB1 affinity, but still high CB2 affinity [174]. The DHA-derived endocannabinoid DHEA and its ω-3 epoxide metabolite lack relevant affinities for either cannabinoid receptor CB1 or CB2 [166,174,178,179]. Thus, there are differences between the different PUFA-derived endocannabinoids and their epoxy-metabolites in terms of CB-receptor selectivity. In particular, EPA-derived EPEA is a general agonist for both CB-receptors and epoxidation changes it to a specific CB2 agonist, while the DHA-derived endocannabionoids lack relevant receptor affinities. This may be significant, since supplementation with ω-3 PUFAs resulted in increased levels of EPEA and DHEA in rat brain at the expense of anandamide [180,181] and because the CB2 receptor is thought to signal anti-inflammatory effects in both peripheral and CNS immune cells [182].

## 3. Omega-3 PUFAs, Membrane Structure and Organization

### 3.1. Differential Effects of EPA and DHA on Lipid Raft Formation

Cellular membranes are not just homogenous mixtures of lipids and proteins but their properties vary in patches. Preclinical findings suggest a crucial role of membrane organization and the associated assembly of signaling proteins in the induction of depression- and anxiety-related behaviors [183]. One kind of patch in the cellular membrane is membrane regions called “lipid-rafts”, which are crucial for cell signaling because key transduction proteins are enriched in these membrane patches [184,185]. For example, the above-mentioned TLR4 is localized in these lipid-rafts and disruption of the latter interferes with TLR4 signaling [185,186]. Preclinical studies provide evidence that the disruption of the TLR4 pathway by PUFA supplementation is not due to changes in gene expression of mediator proteins, but originates at the membrane level [186]. Interestingly, both ω-3 PUFAs EPA and DHA are incorporated in the cellular membrane, but modulate these lipid rafts differently. DHA was shown to increase the size of lipid rafts, presumably through its hairpin-like structure. This is thought to lead to a dispersion of effector molecules such as the TLR4 and its cofactor CD14, which hinders their signal transduction leading to a reduced pro-inflammatory cytokine secretion [185]. In contrast, EPA causes a profound restructuring of both the raft and non-raft membranes [187], but its effect on raft reorganization has not been extensively studied to date. While these experiments were done in macrophages, similar effects presumably occur in microglia and other immune cells [188]. Whether this differential effect of ω-3 PUFAs on the size and lipid composition of the lipid rafts is directly relevant for the pathogenesis of MDDs is, however, currently not known.

### 3.2. Preferential Incorporation of EPA and DHA into Different Types of Glycerophospholipids

When GPCR-agonists, growth factors or cytokines bind to their receptors, they can activate several phospholipase A2 isoenzymes acting on membrane phospholipids to release ω-6 and ω-3 PUFAs [189]. These phospholipases display preferences for certain PUFAs, which possibly depends on the preferential integration of PUFAs into different phospholipids. For example in brain cells, EPA is preferentially incorporated at the SN2 position of phosphatidyl-inositol phospholipids and is released by several phospholipase A2 isoenzymes, amongst which the cPLA2-IVA isoform is quantitatively most abundant [190,191]. In contrast, DHA is incorporated at the SN2 position of phosphatidyl-ethanolamine, phosphatidylserine and phosphatidylcholine, and is released by the A2-phospholipases, iPLA2β and iPLA2γ [192,193,194]. There is evidence that EPA itself enhances the expression of cPLA2 in MDD patients [195] and preclinical animal models suggest that stress and inflammation also lead to increased expression of cPLA2 [196,197]. Hence, it may well be that risk factors like stress and inflammation preferentially promote the release of EPA.

## 4. Discussion

Over fifty controlled studies (see Table 1) investigated the antidepressant effects of ω-3 fatty acids in a range of human conditions, with over a dozen studies focusing on primary MDD. These studies showed that, in particular, patients with moderate to severe depression seem to benefit from ω-3 fatty acids rich in EPA. Rather unexpectedly, controlled studies using DHA-enriched fish-oil preparations or purified DHA failed to demonstrate major antidepressant actions.

This is even more remarkable considering that the mammalian brain has a unique fatty acid composition with high levels of AA and DHA, but remarkably low levels of EPA (see Table 2). In rat brain, for instance, the levels of EPA are 300-fold lower than those of DHA [198]. Since depressive symptoms are assumed to be of central origin, it seems paradoxical that EPA is a more effective antidepressant than DHA with its purported centrally mediated effects on the HPA axis and the neurotropic effects on brain development. In principle, the results from the intervention studies imply that the antidepressant activity of EPA is independent of such mechanisms. Studying the molecular differences that distinguish EPA from DHA (see Table 2) identified three metabolic pathways that may explain the preferential EPA-derived effects. These are the distinct membrane integration and release of EPA by cPLA2, the preferential enrichment of the EPA-derived CYP-metabolome after ω-3 PUFA supplementation, and the higher CB2 receptor affinities of the endocannabinoid EPEA and its epoxy-metabolite 17,18-EEQ-EA.

The first difference between the two ω-3 PUFAs is observed in the integration of EPA and DHA into different phospholipid species in the cellular membrane. After crossing the blood-brain barrier, PUFAs are mainly incorporated into membrane phospholipids. DHA is predominantly inserted at the SN2 position of phosphatidylethanolamine (PE), phosphatidylserine (PS) and phosphatidylcholine (PC) [199,200], whereas EPA is preferentially incorporated at the SN2 position of phosphatidylinositol (PI) [199]. At the same SN2 position of PI, AA is also incorporated. It now seems that EPA competes with its analogous C20-ω-6 fatty acid AA for PI incorporation [201] and that, depending on the PUFA integrated in PI, the downstream signaling events of the specific cell is changed.

For signaling, the PUFAs have to be released from the SN2 position of the phospholipids. EPA has been shown to compete with AA for its release from PI by the PLA2 enzymes and there is an indication that the most abundant PLA2 isoenzyme releasing AA and EPA in brain, cPLA2, is increased by several risk factors for MDD like stress, inflammation and hypertension. This argues for the competition of EPA with AA for eicosanoid production and for the preferential release of EPA from membranes by certain risk factors for MDD upon EPA supplementation. In light of the rather pro-inflammatory eicosanoids produced from AA by the COX, ALOX and the CYP pathways, such a competitive suppression of AA eicosanoids by EPA may well add an additional level of anti-inflammation compared to DHA, and could provide a partial explanation for the “EPA-paradox” mentioned above.

**Table 2:** List of functional differences between EPA and DHA.

Observation	Citation
Last step of DHA synthesis (not EPA synthesis) is in peroxisome	[108,112]
Rate-limiting step in DHA biosynthesis is between EPA and DHA	[202,203,204]
Brain levels of DHA are kept high, while EPA levels are kept low	[193,194,205,206]
Size of lipid rafts is increased by DHA and unaltered by EPA	[185]
DHA is inserted in PE^1^, PS and PC, while EPA is inserted in PI	[199]
EPA and DHA are released from lipids by different PLA2 isoenzymes	[191,192]
COX and ALOX5 metabolize EPA and AA, not DHA	[108,112]
EPA-derived resolvins and DHA-derived resolvins in some cases activate distinct G-protein coupled receptors	[207]
RvE1 reduces, while RvD1 increases platelet aggregation	[157]
EPA, not DHA activates PPARα	[208]
EPA, not DHA increases expression of cPLA2	[195]
EPA, not DHA improves depression	[23,24,25,33]
EPA, not DHA ameliorates cardiac illness	[209]
EPEA and its epoxide, but not DHEA or its epoxide are highly potent CB2 receptor agonists	[174]

Abbreviations. PE: phosphatidylethanolamine, PC: phosphatidylcholine, PS: phosphatidylserine, PI: phosphatidylinositol, PLA2: phospholipase A2, RvE1: Resolvin E1, RvD1: Resolvin D1, PPAR: peroxisome proliferator-activated receptor, EPEA: EPA-ethanolamine, DHEA: DHA-ethanolamine.

The enzymatic metabolism of the ω-3 and ω-6 PUFAs leads to the formation of the wide spectrum of eicosanoids and docosanoids. Different groups have investigated the effect of dietary supplementation with EPA/DHA on the production of these eicosanoids and docosanoids. These studies showed that fish oil supplementation in humans resulted in a large increase in EPA-derived CYP-derivatives and a smaller increase in EPA- and DHA-derived LOX-dependent metabolites in plasma and tissue [168]. The levels of the hydroxy-, epoxy- and dihydroxy-derivatives correlated to the parent PUFA in erythrocytes with a particularly strong correlation between EPA levels in erythrocytes and the concentrations of EPA-derived CYP-metabolites, including 17,18-EEQ [169]. The authors also calculated that the CYP-enzymes metabolize EPA 8-fold more efficiently, and DHA twice as efficiently as AA [168], suggesting that changes upon fish oil supplementation may come from the preferential metabolism of EPA by CYP-isoenzymes. However, whether these EPA- and DHA-derived CYP lipid mediators are also differentially produced in the brain has not been investigated.

Both series of PUFAs are also metabolized to endogenous endocannabinoids involved in appetite control, food intake, energy balance, and several neurological and mood disorders [170]. These endocannabinoids signal through a series of different receptors with the main receptors identified as the endocannabinoid receptors CB1 and CB2. EPA-derived and DHA-derived endocannabinoids were shown to have different affinities for these two endocannabinoid receptors. While the EPA-derived endocannabinoid 17,18-EEQ-EA is a specific CB2 agonist, the DHA-endocannabinoid 19,20-EDP-EA lacks a relevant affinity for CB2-receptors [174]. This seems of interest because the CB2 receptor has emerged as a potential anti-inflammatory target to reduce neuroinflammation [177] and chronic inflammatory diseases [210]. The CB2 receptor is expressed on monocytes, macrophages, dendritic cells, and microglia cells [172,176] and CB2 agonists have been shown to reduce neutrophil, monocyte and macrophage infiltration, to reduce microglia activation and migration, and to inhibit the release of inflammatory cytokines, chemokines, reactive oxygen species and nitric oxide [172,176,177,211]. Since dietary supplementation with EPA and DHA results in an increase in EPEA- and DHEA-levels at the expense of the AA-derived AEA (reviewed by [174]), the formation of, especially, the EPA-derived endocannabinoids and their epoxy-metabolites may alter the level of CB2-receptor stimulation. Such a mechanism may also contribute to the preferential anti-inflammatory and antidepressant profile seen for EPA.

## 5. Conclusions

In conclusion, whilst a satisfactory explanation for the lacking anti-depressant activity of DHA remains obscure, there is a body of indirect evidence that EPA improves depression at least partially by acting as a pro-drug through competition with AA for integration and release from membranes, by its abundant CYP-mediated metabolome, and by the high CB2 receptor affinities of its endocannabinoid EPEA and its epoxy-metabolite 17,18-EEQ-EA. Future research should explore these distinct EPA features as potential novel drug targets for antidepressant drug development.

## Figures and Tables

**Table 1:** Omega-3 RCTs in depression.

Indication	Citation
Major depressive disorders (MDD)	[32,33,34,35,36,37,38,39,40,41,42,43,44,45,46,47,48,49,50,51,52,53,54,55,56,57,58,59]
Depressive episodes in bipolar affective disorders	[60,61,62,63,64,65,66,67]
Depression during or post pregnancy	[68,69,70]
Depression in non-MDD mood disorders (e.g., premenstrual syndrome, dysthymia)	[31,45,49,71,72,73]
Depression in other psychiatric conditions (e.g., borderline personality, self-harm, OCD)	[74,75,76,77,78]
Depression in established schizophrenia	[79,80,81]
Depression in Alzheimer’s dementia/mild cognitive impairment	[46,82,83]
Depression in Parkinson’s disease	[84]
Depression in medical conditions (cerebrovascular and metabolic diseases or cancer)	[85,86,87,88,89]
Depressive features in healthy individuals	[90,91,92,93,94,95,96]

Abbreviations: RCT: randomized controlled trial; OCD: obsessive-compulsive disorder.

## Abbreviations

AA	arachidonic acid
AEA	arachidonic acid-ethanolamide
ALOX	arachidonate lipoxygenase
CB1	cannabis receptor-1
CD14	cluster of differentiation-14
COX	cyclooxygenase
CYP	cytochrome P450
DASS21 score	a 21-item rating scale to quantify depression anxiety and stress
DHA	docosahexaenoic acid
DHEA	docosahexaenoic acid-ethanolamine
EPA	eicosapentaenoic acid
EPEA	eicosapentaenoic acid-ethanolamine
17,18-EEQ-EA	ethanolamine derivative of EPA, where the omega-3 bond is oxidized to an epoxide
ES	effect size
GPR or GPCR	G-protein-coupled receptor
20-HETE	20-hydroxy-tetraenoic acid
MCP1	monocyte chemoattractive protein-1
MDD	major depressive disorder
NLRP	acronym for NACHT, LRR and PYD domains-containing protein
NFκB	nuclear factor-κB
OCD	obsessive-compulsive disorder
PC	phosphatidylcholine
PE	phosphatidylethanolamine
PI	phosphatidylinositol
PS	phosphatidylserine
PLA2	phospholipase A2
PPAR	peroxisome proliferator-activated receptor
PUFA	polyunsaturated fatty acid
RCT	randomized controlled trial
RvE1	Resolvin E1
RvD1	Resolvin D1
ROS	reactive oxygen species
SMD	standardized mean differences
SPM	specialized pro-resolving mediators
TLR	Toll-like receptor
